# Computational and Experimental Approaches to Visual Aesthetics

**DOI:** 10.3389/fncom.2017.00102

**Published:** 2017-11-14

**Authors:** Anselm Brachmann, Christoph Redies

**Affiliations:** Experimental Aesthetics Group, Institute of Anatomy, Jena University Hospital, School of Medicine, University of Jena, Jena, Germany

**Keywords:** computational aesthetics, experimental aesthetics, visual preference, art history, artist identification, style identification, image features, statistical image properties

## Abstract

Aesthetics has been the subject of long-standing debates by philosophers and psychologists alike. In psychology, it is generally agreed that aesthetic experience results from an interaction between perception, cognition, and emotion. By experimental means, this triad has been studied in the field of *experimental aesthetics*, which aims to gain a better understanding of how aesthetic experience relates to fundamental principles of human visual perception and brain processes. Recently, researchers in computer vision have also gained interest in the topic, giving rise to the field of *computational aesthetics*. With computing hardware and methodology developing at a high pace, the modeling of perceptually relevant aspect of aesthetic stimuli has a huge potential. In this review, we present an overview of recent developments in computational aesthetics and how they relate to experimental studies. In the first part, we cover topics such as the prediction of ratings, style and artist identification as well as computational methods in art history, such as the detection of influences among artists or forgeries. We also describe currently used computational algorithms, such as classifiers and deep neural networks. In the second part, we summarize results from the field of experimental aesthetics and cover several isolated image properties that are believed to have a effect on the aesthetic appeal of visual stimuli. Their relation to each other and to findings from computational aesthetics are discussed. Moreover, we compare the strategies in the two fields of research and suggest that both fields would greatly profit from a joined research effort. We hope to encourage researchers from both disciplines to work more closely together in order to understand visual aesthetics from an integrated point of view.

## 1. Introduction

Dating back more than two thousand years ago, aesthetics has been the subject of debates by philosophers and other scholars alike. Defined by the Oxford Dictionary as “the philosophy of the beautiful or of art,” “a system of principles for the appreciation of the beautiful,” and “the distinctive underlying principles of a work of art or a genre” (OED, 2017), aesthetics represents a field of interest that has attracted researchers from diverse scientific disciplines, also outside of philosophy. In 1876, the founder of experimental aesthetics, Gustav Fechner, published his seminal book entitled “Vorschule der Ästhetik” (Fechner, 1876). He believed that the aesthetic appeal of physical objects manifests itself in stimulus properties that can be measured in an objective (formalistic) way. Specifically, he attempted to show that rectangles with an aspect ratio equal to the golden ratio are more appealing to human observers than rectangles having other aspect ratios. Researcher have later raised concerns about the normative role in rectangular preferences (Green, 1995; McManus et al., 2010). Nevertheless, Fechner's scientific (objective) view of aesthetics provided the basis for the newly emerging field of *empirical aesthetics*. In this field, hypotheses regarding the perceived beauty of images, paintings or even every-day objects are proposed and tested experimentally for their validity. This stimulus-driven approach, called by Fechner *aesthetics from below*, was different from the aesthetics that was prevalent in Fechner's time and derived aesthetic principles from superordinate philosophical concepts *(aesthetics from above)* (Cupchik, 1986). Fechner is also credited for conceiving the field of *psychopysics*, which relates human perception to well-defined physical properties of stimuli. By applying this approach to aesthetics, he attempted to relate physical image properties to aesthetic perception in humans. The area of research that has taken up this idea in modern times is *experimental aesthetics*, a subfield of psychology.

Another discipline of natural science that studies aesthetics is *neuroaesthetics*, a subfield of brain research. In this field, modern imaging techniques, such as functional magnetic resonance imaging (fMRI), enable researcher to study the activation of brain regions when human observers view aesthetic stimuli (Cela-Conde et al., 2011; Chatterjee and Vartanian, 2014). This type of research has lead to a better understanding of what neural networks are involved in the human brain when we have an aesthetic experience. Research in neuroaesthetics is beyond the scope of the present review.

In recent years, aesthetics has also been studied using computational methods. In the field of computer science, *computational aesthetics*, a subfield of computer vision, has entered the field of aesthetics. In this area, there have been a variety of different studies on the aesthetics in digital images, for example, using digital reproductions of paintings. The birth of computational aesthetics is often attributed to Birkhoff's book “Aesthetic Measure” (Birkhoff, 1933), although the book does not mention the term itself (for an overview of the evolution of the term, see Greenfield, 2005). In a very mathematical way, Birkhoff proposed a formula for an aesthetic measure *M*, which is a function of *O*, order or reward by a positive tone of feeling, and *C*, complexity or a feeling of effort of attention. Stating that reward should be proportional to effort, Birkhoff concludes that *M* = *O*/*C* best describes their relation.

A definition of computational aesthetics is given by Hoenig (2005), who describes it as “[…] the research of computational methods that can make applicable aesthetic decision in a similar fashion as humans can.” To Hoenig, this definition emphasizes two major aspects: First, the use of computational methods, and second, their applicability to aesthetic decision making. More precisely, Galanter (2012) discusses how computational aesthetics is concerned with both, “the creation and evaluation of art using computers.” He argues that the creation of art necessarily requires evaluation and gives the example of an artist, who, while learning about aesthetics and gathering experience, evaluates art created by others. When creating artworks himself, micro-evaluations help the artist guide his own creative process. Upon finishing his creation, the artist gains new insights about his art in a final evaluation of the created piece. Given the importance of the evaluation process, we will focus on it in the present review. As pointed out by Stork (2009a), the computational analysis of paintings has several advantages compared to an analysis carried out by human experts. For example, a computational analysis can pick up very subtle relationships that may escape the attention by human observers; moreover, computational methods are objective in nature and are potentially non-exhaustive in the amount of detail analyzed (e.g., every single brushstroke in a painting).

The aim of the present review is to provide an overview of recent developments in the field of computational aesthetics and to point out its potential relevance for research in experimental aesthetics and vice versa. Our goal is to boost the awareness of researchers in experimental aesthetics for the wealth of data that computational aesthetics has generated in recent years. We would also like to inform scientists in computational aesthetics about some basic concepts and results from experimental aesthetics. Our review thus outlines a possible link between research on the objective (physical) properties of visual stimuli and experimental studies that take into account the subjective responses of humans to aesthetic stimuli, as originally proposed by Fechner. Specifically, we focus on the evaluation of visual images (photographs or digitally reproduced artworks) and the analysis of image properties. Important areas of research will be referenced and exemplary works will be presented, without striving for completeness. Topics include the prediction of ratings of photographs and paintings, the classification of images regarding their artist or style, computational methods for problems in art history, and, finally, the investigation of statistical properties of aesthetically pleasing images and artworks.

## 2. Computational aesthetics: algorithms and applications

Computational aesthetics is approached from different points of view. All articles reviewed here somehow deal with aesthetics in the form of photography and paintings and are motivated predominantly by producing applications and testing or improving algorithms. Accordingly, one of the tasks that is often pursued in computational aesthetics is to develop algorithms that allow to predict aesthetic ratings of photographs. Such algorithms have direct applications. For example, in online photo communities (for example Flickr, Photo.net, etc.), they can be used to select photographs of high aesthetic quality and discard snapshots that users would rate low. On a more commercial side, such systems are used for retrieving and licensing high-quality photographs from the internet for their use as stock photographs. Another possible application is to install such algorithms in industrial cameras and smartphones, which identify high-quality images in the split of a second. As we will show in the present article, there has been a tremendous success in building such systems.

The prediction of ratings is just one possible application among many, where computers can make decisions regarding aesthetics. Computational methods have also been successfully applied to problems in art history, such as content analysis of paintings, forgery detection, or detection of a painter's influence. These applications will also be reviewed in the following sections.

### 2.1. Prediction of ratings

One major trend in computational aesthetics is to predict ratings of image quality or aesthetic appeal. Possible applications of this technology are improved cameras, which automatically select the most appealing photos among many, optimization of advertisements for their aesthetic value, or even talent scouting in photo-sharing communities. In the early days of computational aesthetics, researcher followed the then popular practice to design features explicitly for a given task. In order to predict the aesthetic appeal of a given image, researchers determined in how far different photographic principles, like composition according to the rule of thirds or depth of field, were followed in images. They quantified these principles by expressing them numerically, either as binary or continuous values, called features. Features can be either local, describing only pixels or patches and their immediate neighborhood, or they can be global and describe properties of the image as a whole. Global features seem especially suitable to describe artistic photographs or artworks because concepts such as artistic composition refer to the relation between pictorial elements across the image. Another difference can be made concerning the level of abstraction: Low-level features describe basic features, such as colors and edges, while high-level features can describe more abstract image content. The features can then be used to train a classifier on a dataset of images so that it can learn to predict ratings given by humans. This goal is achieved by mathematically describing the relation between the subjective scores and the feature set. Popular choices for classifiers are, for example, Bayes classifiers, Decision Trees, or Support Vector Machines (SVMs). This approach will be presented in more detail in section 2.1.1. In recent years, computational aesthetics has gone from designing features by hand to using generic features that have been developed for other purposes in computer vision. This development has reached a pinnacle with the development and widespread use of Deep Neural Networks. Approaches using generic features will be discussed in section 2.1.2.

#### 2.1.1. Hand-crafted image features

One of the first attempts to measure aesthetics in an image was published by Tong et al. (2004), who proposed a method to distinguish between photographs taken by professional photographers and photographs taken by non-expert (home) users. They used a set of low-level features that describe blur, contrast, colorfulness and saliency, and combined it with general purpose low-level features that capture texture, shape and energy in the frequency spectrum, by using difference-edge histograms. In total, they proposed 21 different features which added up to 846 dimensions. After reducing the dimensionality, they reported classification results comparing Boosting, an SVM and a Bayesian classifier, which performed best.

Using another set of low-level features, Datta et al. (2006) build a classifier for distinguishing images of high aesthetic appeal from other images, as rated by the community of the popular photo-sharing website Photo.net. Overall, the authors collected 3,581 different images and split them into two classes according to their aesthetic rating by the users of the site (low and high rating). They explicitly stated that their goal was not to build the best-performing classifier, but rather to be able to draw conclusions from the best performing features. Their choice of features was based on common intuition, rules of thumb in photography and trends that they observed for the ratings of the collected images. In total, they proposed a set of 56 different features, containing basic ones, such as colorfulness, saturation, hue, size and aspect ratio, as well as adherence to the rule of thirds. The features were selected as follows: First, the authors used a one-dimensional SVM to find the features with the most discriminative power and selected the top 30. Starting with an empty features set, they then iteratively added those features that improved the classification the most. As a result, they found that average hue, average pixel intensity as well as a saturation-based rule of thirds measure contributed the most to the aesthetic value of an image, as rated by human observers.

Ke et al. (2006) designed a system to distinguish between high-quality professional photographs and low-quality snapshots. They reference the work of Tong et al. (2004) but criticize their black-box approach, which prevents them from gaining any insight into why some photos are better than others, although the system by Tong and colleagues performed well for the task. Ke et al. (2006) therefore chose an approach similar to the one by Datta et al. (2006) and designed a set of features that capture image quality. They based their choice of the features on interviews conducted with photographers. Their feature set contained the spatial distribution of edges, color distribution, hue count and blur as well as contrast and brightness. For classification, they used a naive Bayes classifier and tested their system on images that were downloaded from a photo contest website. The blur feature turned out to be the most discriminative metric.

Luo and Tang (2008) extracted very simple features that captured lighting, simplicity, composition or color harmony, based on the subject region and the background of an image. They reported an improvement of classification upon Datta et al. (2006) and Ke et al. (2006) and contributed this success to the distinction of foreground and background, while the previous methods computed their features on the image as a whole.

Besides focusing on low-level features as provided by Ke et al. (2006) and Dhar et al. (2011) also integrate high-level attributes in their system in order to predict aesthetic value and interestingness. According to the authors, high-level attributes define characteristics of images as humans would describe them, and can be classified into compositional attributes (like the rule of thirds), content attributes (like the presence of people) and sky illumination attributes. Dhar et al. (2011) reported improved performance compared to the approach by Ke et al. (2006).

Although the general focus of aesthetic quality assessment in computational aesthetics is on the prediction of ratings of photographs, a few researchers have also proposed methods for quality assessment of paintings. Li and Chen (2009), for example, propose a total of 40 features that capture color, brightness and compositional characteristics of a paintings. Using these features, they use a Bayes classifier as well as AdaBoost on a binary task to predict whether a painting received high or low rating scores. In their work, they provide a detailed discussion of the importance of the individual features.

What all these approaches have in common is that a combination of multiple features is used to predict aesthetic ratings. While this has proven successful for automated aesthetic decision making, there are a number of problems that preclude a deeper understanding of the role of individual features in these decisions. First, because the features are not necessarily independent of each other, it would require more sophisticated statistical methods to extract the influence of each of them. Second, the experimental conditions, under which ratings are obtained in most of the above-mentioned studies, are unknown, unspecified or variable (for example, with regard to the size of the stimuli on the retina, the brightness of the stimuli, contrast settings of the monitors, background illumination, sequence of stimulus presentation etc.,). Third, the rating by users of internet platforms often remain anonymous which precludes any specification of their personal characteristics (sex, age, cultural background etc.,). All these factors might influence the results or introduce artifacts.

In experimental aesthetics, some of the features used in the above combinatorial approaches have been isolated and studied in psychological experiments under well-defined experimental conditions (for a survey of such studies, see section 3).

#### 2.1.2. Generic image features

Generic image features are features that are not explicitly designed for the prediction of image aesthetics, but rather for other popular research topics in computer vision, like object detection and classification, scene understanding, or image retrieval. An example of such features are the SIFT descriptors (scale-invariant feature transform; Lowe, 2004), which were originally designed for feature matching and image stitching. SIFT encodes edge orientations in gray-scale images as a vector (for more recent image descriptors, see Canclini et al., 2013).

The first study to model aesthetic ratings based on generic image features was published by Marchesotti et al. (2011). They used SIFT descriptors together with a color descriptor, motivated by the assumption that aesthetic properties, such as the presence of sharp edges or the saturation of colors, can be described implicitly by these kind of features. The authors chose a Bag-Of-Visual-Words and a Fisher-Vector representation in order to represent prototypical patches for aesthetic and non-aesthetic photographs. As a result, they reported an improvement in classification rates for high-quality and low-quality images, compared to the methods by Datta et al. (2006) and Ke et al. (2006) who used hand-crafted features (see section 2.1.1). While hand-crafted features allow to quantify which feature contributes the most to an aesthetic rating, this interpretability is lost with generic features. Here, conclusions can only be drawn by a comparison of the images that are rated high or low by the model because the features of the model are not deliberately designed to capture known properties of aesthetics, but they rather hide their relation to them. For example, Marchesotti et al. report that all blurry and low-resolution images were rated low in his model, whereas images that displayed foreground objects with sharp edges on out-of-focus backgrounds were rated highly. Moreover, highly-rated images had a dominant color or used complementary colors in their palette; if too many colors were present, images received low scores in general. On the same dataset, Murray (2012) used a low-level contrast model that was originally developed for saliency estimation and showed that it can also be applied to predict aesthetic preferences.

In recent years, deep learning models, in particular Convolutional Neural Networks (CNNs), have started to conquer many subareas in the field of computer vision and artificial intelligence. Although the basic idea of CNNs has already been proposed more than three decades ago (Fukushima, 1980; Lecun and Bengio, 1995), only recently, progress in computing technologies and the availability of huge datasets for training have helped to restore the interest in using CNNs for image processing (Krizhevsky et al., 2012; Simonyan and Zisserman, 2014; He et al., 2015; Huang et al., 2016). CNNs learn a hierarchy of filters, which are applied to an input image in order to extract meaningful information from the input. The training is done using backpropagation, a supervised training algorithm, in which the current output of a network is compared to a desired output. Filter parameters of the network are changed according to their contribution to the current error. When used on a large training set of images, CNNs tend to learn features that resemble Gabor-like edge detectors and color-opponent filters at lower layers of the CNNs. These features are akin to neural responses in the early mammalian visual system. On higher layers of the CNNs, features capture more abstract image content by integrating the lower-layer features (Yosinski et al., 2015). Different open-source implementations exist, which also include a variety of models that were pretrained for object or scene recognition. Their availability enables researchers to either retrain networks that already work well for recognition tasks (a process called fine-tuning), or to use features from pretrained models without any further modification.

CNNs have been applied to the task of rating image aesthetics. Lu et al. (2015) trained a two-column deep neural network simultaneously on global and local views of photographs in order to predict their aesthetic rating class (high or low). The authors motivated their architecture by the observation that the aesthetics of an image is influenced by local cues, such as sharpness, as well as global cues, which capture compositional aspects. They evaluated different cropping strategies for the local image view and report a higher accuracy in the prediction of image aesthetics than reported for previous approaches on the same dataset (Murray et al., 2012).

Dong et al. (2015) applied the AlexNet architecture presented by Krizhevsky et al. (2012), which was trained on 1.2 million images to discriminate between 1,000 different object categories. They used the features of the top convolutional layer, which are computed on the entire image, as well as on five local crops, and trained an SVM on the concatenated features. They improved upon the results by Marchesotti et al. (2011) by a margin of about 10%. Interestingly, their approach did not explicitly use features trained in the context of an aesthetic evaluation, but rather for object recognition, so that the decision whether an image was rated as highly aesthetic or not seemed to rely more on image content than on image form.

Denzler et al. (2016) proposed to use CNNs as model of perception for research in aesthetics. They trained the AlexNet model (Krizhevsky et al., 2012) on different datasets to experimentally evaluate how well pre-learned features of different layers are suited to distinguish art from non-art images using an SVM classifier. They report the highest discriminatory power with a Network trained on the ImageNet dataset, which outperforms a network solely trained on natural scenes.

Kao et al. (2016) proposed a multi-task learning approach, in which a CNN was trained to simultaneously assign semantic and aesthetic labels. They explored different network architectures and showed that a network trained to recognize semantic labels in addition to the aesthetic class outperforms a network trained solely to recognize the aesthetic class of an image. This finding is compatible with the role of both content and form in psychological models of aesthetic experience (see below).

Nowadays, deep neural networks have largely replaced the conventional approach of designing features deliberately in order to reflect aesthetic concepts that derive from human intuition. They outperform the conventional approach easily and have a number of additional advantages: (1) Deep neural networks learn features that are important for aesthetic evaluations automatically, provided that a dataset is big enough. (2) They can combine local image properties, such as sharpness or blur, with global properties, such as composition or color harmony. (3) They can even take into account abstract features, such as image content, without the explicit design of such features by humans. (4) Last but not least, deep neural networks are able to learn image properties that humans may not even be aware of. Such properties include unspecified compositional rules that are employed intuitively by photographers and painters (Bell, 1914; Arnheim, 1954; Redies, 2007, 2015).

While deep learning models are state-of-the-art in aesthetic image evaluation, their success comes at a cost. At present, the understanding of deep features and how they work in object or aesthetic recognition lacks behind. Although there have been attempts to analyze what deep neural networks actually encode at higher layers (Yosinski et al., 2015), we are far from understanding the success of deep learning in any significant detail. For applications in aesthetic image evaluation, it may be sufficient to simply build systems that closely match human perception in deciding whether an image is considered to be beautiful. However, for researchers who want to learn more about aesthetics *per se*, the limitations of deep learning models are particularly obvious. With handcrafted features, it is easy to draw conclusion about which features contribute to the aesthetic value of an image. Deep neural networks and generic features basically represent a black-box approach that lacks this kind of interpretability. Nevertheless, if we can develop tools to understand deep representations in the future, the drawback of deep learning approaches may eventually turn out into an asset for understanding aesthetics. Such a more profound understanding would also require that deep learning be better explainable in terms of actual neural mechanisms. Although some recent studies lead in this direction (for example, see Brachmann et al., 2017), an abundance of questions remains.

### 2.2. Other classifications of images

Besides the prediction of visual preference, there has been another trend in computational aesthetics, which tends to be more focused on artworks than on photography. In this trend, images are not classified according to their aesthetic appeal, but with respect to the correct identification of the painter or the artistic style, an undertaking which is usually performed by art experts. From a methodological point of view, the identification of painter and style are related tasks that often go hand in hand. However, in the early days of computational aesthetics, the identification of the artist who created a given painting (Cezanne, Vermeer, Rembrandt, etc.,) was more popular. More recently, there seems to be a shift to the prediction of the style (Realism, Impressionism, Cubism, etc.,), as works from more and more art collections become digitized and available on the web. These open-source collections enable researchers to easily collect the huge number of images that are needed in order to train and test algorithms. Possible applications for such methods are recommender systems for online art markets or the more precise description of the stylistic singularities of particular artists.

#### 2.2.1. Artist identification

Using a Naive Bayes Classifier, Keren (2002) computed Discrete Cosine Transform (DCT) coefficients on an image and identified the painters of art images (Rembrandt, van Gogh, Picasso, Magritte, Dali) by using a voting scheme, where each 9 × 9 block of an image is assigned the style of an artist. A majority voting for an image yielded the final result and the authors reported an accuracy of 86% for choosing the correct painter. Widjaja et al. (2003) focused on nude paintings and used color of skin in order to identify the artist. They trained an SVM on color profiles of patches extracted from images of four different painters (Rubens, Michelangelo, Ingres, and Botticelli) and reported a rate of correct identifications of 85%. Li and Wang (2004) proposed a system for artist identification based on wavelets and a Multiresolution Hidden Markov Model and tested their approach on a dataset of grayscale Chinese ink images that contained works by five different Chinese artists. Besides the classification of paintings regarding their artist, they found that their modeling approach can also be used as a measure of similarity. To recognize the artist of an image, Lombardi (2005) proposed a system that used a set of low-level features for intensity, edge information, spatial frequency information, as well as a new feature that captured color. Shen (2009) combined a set of global visual features (color, textures, shape) and local visual features (Gabor wavelets) and reported an identification accuracy of 69.7% when distinguishing 25 classical Western painters in a dataset that included Caravaggio, Rubens, Vermeer, and van Gogh. For classification, they used an RBF neural network. Khan et al. (2010) automatically predicted painters (Ingres, Matisse, Monet, Picasso, Rembrandt, Rubens, Titian and van Gogh) by using a Bag-of-Visual-Words approach. They computed SIFT descriptors, as well as color name descriptors and trained an SVM on a dataset which consisted of 40 images each of the eight artist (320 images total). They report an accuracy of 62% for the combination of color and shape features. Condorovici et al. (2013) used a dataset of 1,896 paintings by 15 different artist (including Pollock, Rembrandt, Cezanne, and Magritte), from which they extracted low-level features like an RGB color histogram and edge information by Gabor filters. The authors experimented with eight different classifiers, among which multi-class logistic regression yielded the best results. Cetinic and Grgic (2013) extracted three types of features, namely image-intensity statistics, color-based features, and texture-based features and used a multi-layer perceptron with one hidden layer; they reported a 75.3% accuracy of identifying the correct one among 20 painters.

Overall, it is difficult to compare the performance of the different methods for artist identification because a common database, on which results could be reported and compared to others, is lacking to date. Condorovici et al. (2013) addressed this problem by comparing different methods to their guessing baseline. However, this approach may give an advantage to researcher who select painters who are more diverging to begin with. For example, it may be harder to distinguish an impressionist painting by Claude Monet from one by Paul Cezanne, than to distinguish an abstract drip painting by Jackson Pollock from a surrealist painting by René Magritte.

In summary, the most popular choices for features that are used for the classifiers include a measure to capture texture or spatial frequency, edge histograms for shape detection and histograms for color analysis; all these features are low-level and do not describe image content.

More recently, classification studies in other areas of research no longer rely on one classifier, but report results for a set of different classifiers that are studied in parallel. A popular choice for this type of analysis is the Weka data mining software (Hall et al., 2009).

#### 2.2.2. Style prediction

To predict art styles in various sets of artworks, different approaches have been used. Gunsel et al. (2005) trained an SVM classifier in order to discriminate among five painting styles (Classicism, Impressionism, Cubism, Expressionism, and Surrealism) as well as between twelve different painters. They proposed a system that computes a 6-dimensional vector of low-level features including brightness and gradient information of an image as well as statistics of the gray-level histogram. This system allows a user to query the system for similar paintings of unknown style. For painter and art movement classification, the authors report a high accuracy with a low number false positive results. A different approach was taken by Jiang et al. (2006) who designed a way to retrieve traditional Chinese paintings and then classify them into one of the two styles, *Gongbi* (traditional Chinese realistic painting) or *Xieyi* (freehand style). For this task, they used low-level features, which captured color, texture and edges. With a classifier that combined a decision tree and SVMs, they obtained accuracies that are suitable for practical purposes.

Wallraven et al. (2009) asked participants to group images from 11 different art periods (e.g., Gothic, Renaissance, Classicism, Surrealism and Postmodern Art) and different artists into self-selected categories. The resulting categories of artworks corresponded well with the canonical art periods. The authors then computed several low-level features of the images (e. g. raw pixel values, color histograms, frequency, or a GIST descriptor; Oliva and Torralba, 2006) and tested how well the features described the clustering into different art periods. The authors found a low correlation between their set of low-level features and the grouping into art periods and concluded that humans rely more on higher-layer properties. Siddiquie et al. (2009) used multiple kernel learning in their approach and chose texture, histograms of gradient orientations (HOGs), color, and saliency as their features to discriminate between seven different styles (Abstract Expressionism, Baroque, Cubism, Graffiti, Impressionism and Rennaissance). Zujovic et al. (2009) chose five different genres (Abstract Expressionism, Cubism, Impressionism, Pop Art, and Realism). As features, they used steerable filters as well as edge information extracted by a canny edge detector. For color, they calculated HSV histograms and used their bins as features. The classification was done with several different classifiers and the authors reported a best overall accuracy of 69.1% for the AdaBoost classifier. Shamir et al. (2010) classified paintings of nine artists of different genres (Impressionism, Surrealism and Abstract Expressionism) and reached an accuracy of 91.0% in style classification by using a set of features that contained frequency statistics, edge information and color information. Čuljak et al. (2011) focused on texture and color features, stating that such features are closely related to the way humans perceive artworks. As genres, they chose Realism, Impressionism, Cubism, Fauvism, Pointillism and Naïve Art. They tested a range of classifiers and reported best results for an SVM, reaching 60.2% accuracy. Ivanova et al. (2012) used various MPEG-7 descriptors in order to distinguish different art styles. In their experiment, they noted that color features were better suited than texture features for distinguishing between art styles and artists. Condorovici et al. (2015) reported that key to a better accuracy in style discrimination is to let features be inspired by human perception. Accordingly, they used luminance and features that detected shape, texture, edges and color. A total of eight genres was selected for style classification in their study. Like other authors, they tested a set of classifiers and reached best results with an SVM, outperforming their predecessors.

While all articles mentioned above used low-level features, which capture formal aspects of paintings, results from Arora and Elgammal (2012) first indicated that semantic features are also important for style classification. The author compared different features and reported the best results for an SVM trained on classeme feature vectors (Torresani et al., 2010), which represent an image as combined classification scores for many weak classifiers that were trained on low-level descriptors.

Beginning with the work of Krizhevsky et al. (2012) and due to the renewed interest in deep neural networks, these models have also been applied to style prediction. Karayev et al. (2013) used a relatively large dataset of 100K images together with color features, GIST descriptors, saliency, meta-class features (Bergamo and Torresani, 2012) for image content, as well as DeCAF features (Donahue et al., 2014), which are activations of higher layers of CNNs that encode image content rather than image form. They additionally trained a classifier for content features on the categories of animals, vehicles, indoor objects and people. For 25 different painting styles, they reached a mean accuracy of 47.3% with all features in combination. Other than painting style, they also reported results for photographic styles in their article. One of their main conclusions is that style is highly dependent on content. Another approach that also relied on DeCAF features can be found in Bar et al. (2014). These authors reported that a combination of DeCAF features and PiCoDes features (Bergamo et al., 2011), a binary descriptor, which incorporates several low-level descriptors, shows the best performance in style recognition.

Saleh and Elgammal (2015) used the object labels that were produced by the networks proposed in (Krizhevsky et al., 2012) as a feature to discriminate the artist, the style and the genre of roughly 80K paintings. They concluded that classemes (Torresani et al., 2010) are the best way to represent artist, genre, and style-specific properties for discrimination. Tan et al. (2016) conducted several experiments regarding painting style, genre, and artist discrimination and used the architecture proposed by Krizhevsky et al. (2012). They fine-tuned a model that was trained on the ImageNet (Deng et al., 2009) dataset for object recognition, trained a model from scratch, and also tested SVM classifiers on deep features. Interestingly, the fine-tuned model yielded the best results in all tasks and even outperformed the model that was trained from scratch.

Painter and style prediction go hand in hand. In the early days, hand-crafted features that captured the same type of image properties were equally suitable for both tasks. With more and more image data becoming available for training, style prediction can now be trained and tested on exceedingly large sets of images and collections of style categories can be expanded with ease. For painter identification, this is not necessarily the case because, for most artists, only a relatively limited number of paintings are available for training deep networks. As another complicating factor, many artists changed their style during their lifetime. For example, several abstract artists started their career with realistic paintings (for example, Wassily Kandinsky, Piet Mondrian, and Jackson Pollock). As a result, training deep neural networks for painter identification will likely remain more difficult than for style prediction.

For style prediction, the availability of huge collections of digitized artworks will open new possibilities for researchers who will use machine learning methods in the future. For example, popular and widely used datasets of paintings, such as the databases of the Google Art Project and WikiArt (formerly WikiPaintings), contains several thousands of annotated artworks.

As outlined for rating prediction (section 2.1), deep features are getting more and more popular for style prediction and increasingly replace hand-crafted features because they are capable of representing semantic information also. For example, Chiaroscuro style paintings often depict indoor scenes and people, while Impressionist paintings frequently display landscapes. Therefore, deep features do well on style prediction and prove to be more powerful than low-level features that focus on image form only. On the other hand, as with the prediction of ratings, interpretability is not as high as it has been with purposely designed features.

Although the vast area of computer-generated artistic images is beyond the scope of the present review, we would like to point out that deep models have boosted recent developments in this area that harbor a large potential for understanding aesthetics. Gatys et al. (2016) proposed an algorithm that can transfer the style of any image to another, by matching the statistics of the gram matrix of lower-layer features, as well as image content that is represented at higher layers. They demonstrated that arbitrary images can be redrawn in the style of famous paintings from Van Gogh or Picasso. More recent generative models (Generative Adversarial Networks [GANs]; Goodfellow et al., 2014) are even capable of matching the style of entire collections of artworks, as shown by Zhu et al. (2017), who used collections of paintings by Monet, Cezanne and Van Gogh to redraw landscape photographs to match the respective painter's style. While GANs are advanced methods that originate in Machine Learning, other methods like the approach by Malo and Simoncelli (2015) focus more on using physiologically plausible architectures to generate images with similar textures. This latter approach is likely to have more explanatory power because it makes use of mathematical tools that are more directly related to findings from vision science.

### 2.3. Other applications

In the previous sections, we described computational methods to predict ratings and to discriminate between paintings by different artists and art styles. Most of these methods rely of the perceptual distinctness of different types of artworks. However, art has also been studied from other perspectives. In the present section, we review computational methods that can provide useful help in solving questions relevant to art history as well as art forgery detection. Some of these methods aim to discriminate rather subtle differences between artworks that may not even be apparent to the human eye.

For a review on earlier methods, see Stork (2009a). A more recent overview is given in Spratt and Elgammal (2014), who list different applications and publications of computational methods for art analysis, including semantic annotation of artworks, ordering of paintings by creation date, or the detection of similarities in paintings and artists in order to reveal mutual influences between artists.

#### 2.3.1. Art history

Among the methods that address art historical questions, we can discern two areas of interest. First, some researchers have developed computational methods to study artistic technique. Second, the influence of a painter on the style of other artists has been studied.

Criminisi et al. (2002) developed methods for investigating the perspective and the reconstruction of the 3-dimensional space from realistic paintings. This information can help art historians to answer spatial questions like, for example, to determine the height of people or objects that are depicted in paintings. In another study, Criminisi and Stork (2004) analyzed inaccuracies in the perspective cues in a painting by Jan van Eyck and demonstrated that is it unlikely that the painter used optical aids like mirrors during the creation of the painting “Portrait of Arnolfini and his wife.” Stork and Johnson (2006) applied a technique that was originally designed for detection of tampering in photographs, in order to localize light sources in paintings. They presented such an analysis for Georges de La Tour's painting “Christ in the carpenter's studio.” Based on their findings, they rebutted the claim that the light source of the depicted scene lays outside the painting, which could have been an indication of the use of optical aids as well. Papaodysseus et al. (2006) investigated the use of stencils in late Bronze Age wall paintings by applying a Hough Transform (a method for finding instances of mathematically defined shapes in images), and identified a set of stencils that were likely used during creation of the wall paintings. Kim et al. (2014) propose statistical measures to quantify the usage of individual colors, their variety in a painting, and the roughness of the brightness of a painting and report significant differences for different art periods. Berezhnoy et al. (2005) studied color and texture features in paintings by van Gogh. They confirmed that the painter increasingly made use of opponent colors later in his lifetime. Later, Berezhnoy et al. (2009) proposed a method for aiding art experts in automatically extracting the orientations of brushstrokes in a painting.

The study of a painter's influence on other artists, which can be investigated by detecting similarities between images, is a popular topic of research in computational aesthetics. Bressan et al. (2008) used SIFT features and local color statistics to compute similarities between images based on a Fisher Kernel representation of the images. Shamir and Tarakhovsky (2012) used a set of 4,027 features that represented many different aspects of visual appearance (e.g., shape, texture, color) and computed a phylogeny, which shows distinct clusters for classic artists like Vermeer or Rembrandt and for modern artists like Jackson Pollock, Marc Rothko, or Wassily Kandinsky. Wang and Takatsuka (2012) extracted color and composition features, which allowed them to classify Renaissance, Impressionist and Postimpressionist paintings. Furthermore, they applied hierarchical clustering in order to identify relationships among artists and demonstrated that they can detect influences of preceding art periods on Picasso's works. Abe et al. (2013) proposed a framework for determining artistic influences based on the semantics of images. By using classeme features to compute distances between images (Torresani et al., 2010), they succeeded in identifying novel cases where one artist influenced another, which had not been considered by art historians before. Elgammal and Saleh (2015) approached the problem of assessing creativity in terms of the originality of an artwork and represented influences and originality as a graph. Relying on classemes for subject matter and GIST features for compositional aspects, they computed a creativity score for each painting in comparison to contemporary artworks.

#### 2.3.2. Forgery detection

Another example where computational methods can help art historians is in the detection of forgeries, which is a problem closely related to artist identification. In artist identification, the works of an artist are identified among many others that usually possess rather different characteristics, which are often obvious even to laymen. However, when detecting forgeries, any differences may no longer be as easy to spot so that the task may be difficult even for art experts. Both approaches aim at identifying unique features of an artist, but an algorithm, which works well for artist identification, may not work as well for authentication and vice versa.

For example, Lyu et al. (2004) performed a wavelet decomposition of eight works attributed to the Renaissance painter Pieter Bruegel the Elder and five imitations of his work. From the wavelet statistics, they extracted a feature vector for subimages of each image and performed authentication by measuring distances between these high-dimensional points. They found that imitations of Bruegel's works differ significantly from authentic paintings. In another application of their technique, they solved the problem of “many hands.” Here, art historians are interested in how many different painters contributed to one particular painting. Using their method, they were able to identify at least four different painters for face depictions in an image attributed to Pietro Perugino, a notion that is shared by art historians. Polatkan et al. (2009) introduced a new dataset of images that included originals and purposely copied paintings. Using the parameters of a Hidden Markov Model trained on wavelet coefficients, they succeeded in discriminating the copies from the originals. Li et al. (2012) studied the brushstrokes of paintings by Vincent van Gogh and used them for comparison with contemporaries and forgeries, as well as for dating different periods of van Gogh's work. Johnson et al. (2008) summarize different approaches by three research groups for discriminating between 82 original van Gogh paintings, 6 non-original works, and 13 paintings of questionable authorship. All approaches are based on a wavelet decomposition of the images.

The work of American painter Jackson Pollock has received particular interest from the scientific community. Taylor et al. (1999) performed a fractal analysis of the artist's drip paintings and found that the fractal dimension, computed using a box-counting approach, increased over the artist's lifetime. The authors suggested that this method could be used for authenticating or dating individual works by the artist. Taylor's approach was criticized by Jones-Smith and Mathur (2006), who showed that they could easily generate images that had the same fractal properties albeit not being similar to Pollock's paintings in their aesthetic value. Stork (2009b) later defended Taylor and colleagues and argued that, while one feature in isolation may not be sufficient for the analysis, a combination of multiple fractal measures can provide useful information. Shamir (2015) used a set of features from biological image analysis (Shamir et al., 2008) and reported an accuracy of 93.0% in discriminating between original and non-original drip paintings.

Hughes et al. (2010) applied a sparse coding scheme in order to compare authentic Bruegel paintings with works by imitators. They demonstrated that their technique can be used to discriminate between authentic and non-authentic Bruegel drawings. Olshausen and DeWeese (2010) suggested that the methods of detecting forgeries brought forward by Hughes et al. (2010) could be useful not only in learning styles of particular artists but also for using these statistics to generate novel images. Montagner et al. (2016) proposed a system for forgery detection of paintings by the Portuguese painter Amadeo Souza-Cardoso. In their approach, they combined a brushstroke analysis using SIFT features on RGB images and an analysis of the pigments in the painting by hyperspectral imaging. Using a dataset of 12 images, among which one was not painted by the artist, they successfully determined the authenticity of the original paintings.

In summary, computational methods can provide support for art historians who study individual paintings or artists. Computational methods have aided art historians in multiple ways, for example by enabling them to detect the use of practical aids like stencils or projectors in the creation of an artwork. Furthermore, telling forgeries from originals as well as the dating of an artist's work can be improved with the help of algorithmic approaches. Other applications are the exploration of hitherto unknown influences between artists.

## 3. Experimental aesthetics: investigation of specific image properties

In experimental aesthetics, researchers are not primarily interested in reaching automatic decisions that mimic human aesthetic judgments. Rather, the goal is to find out on what grounds aesthetic judgement are made by human observers and what their biological basis and evolutionary purpose might be. In other words, applications are not the focus of research, but rather a better understanding of aesthetic experience (Berlyne, 1974; Cela-Conde et al., 2011; Chatterjee and Vartanian, 2014; Shimamura, 2014). Before proceeding to concrete examples, we will briefly review some key concepts in experimental aesthetic research.

### 3.1. Basic concepts in experimental aesthetics

It is generally agreed that aesthetic experience is a highly complex phenomenon and involves at least three key domains (perception, cognition and emotion), which are realized at multiple levels of human social organization (universal, cultural and individual) (Jacobsen, 2006; Marković, 2012; Chatterjee and Vartanian, 2014; Redies, 2015).

To a large extent, perception represents bottom-up processing of visual information. Perceptual mechanisms are thought to be universal among humans and are likely to have their origin in the evolution of the human visual system. Whereas it is self-evident that any information associated with a visual stimulus must be processed by the visual system in order to be perceived, it is still a matter of debate whether there are specific mechanisms that mediate the perception of aesthetic (or beautiful) stimuli at lower or mid-levels of visual processing.

On the one hand, it has been demonstrated that visually pleasing images are associated with specific image features that can be measured by objective means. Because artworks of different styles, cultures and artists differ in their content, these common image properties reflect formal characteristics of images (*significant form*; Bell, 1914). Possibly, these stimulus properties elicit a particular state of neural activity in the visual system (*resonance*; Taylor et al., 2005; Redies et al., 2007b) or induce the activation of a specific (beauty-responsive) neural mechanism in receptive individuals (Redies, 2015). This specific activation can be thought of as the correlate of visual preference or, more specifically, of the perception of beauty in images.

On the other hand, it has been argued by some modern philosophers, art critics, psychologists and neuroscientists that any visual stimulus can elicit an aesthetic experience, as long as it is presented in an appropriate cultural context. Followers of this cognitive hypothesis often reject the notion that there are objective and universal stimulus properties that characterize aesthetic stimuli. Instead, they emphasize the role of the art-historical context of artworks, the intentions of the artists, conceptual issues, the expertise of the beholder, the status of the artwork and other culturally determined factors (Danto, 1981; Leder et al., 2004; Zeki, 2013; Gopnik, 2014). These factors are, by definition, not universal and do not persist over time, because cultural conditions change perpetually; they reflect cognitive (predominantly top-down) mechanisms in the human brain and relate more to the content and context of artworks than to their form. However, perceptual (sensory) and cognitive factors are not mutually exclusive in aesthetic appreciation; several researchers have included combinations of both types of factors in their models of aesthetic experience (for example, see Jacobsen, 2006; Locher et al., 2007; Marković, 2012; Chatterjee and Vartanian, 2014; Kozbelt and Kaufman, 2014; Shimamura, 2014; Redies, 2015).

Individual experiences also play an important role in aesthetic experience, both in terms of short-term adaptation to the beauty of visual stimuli and in long-term processes, such as familiarization and the acquisition of knowledge about art. Interestingly, interindividual differences have been found even in the preference for basic stimulus properties, such as stimulus complexity (Bies et al., 2016a; Güçlütürk et al., 2016; Lyssenko et al., 2016; Spehar et al., 2016), color (Mallon et al., 2014; Palmer et al., 2016), or the preference for the aspect ratio of rectangles (McManus et al., 2010). Last but not least, the emotions of the beholder also play an important role in aesthetic appreciation (Leder et al., 2004, 2014; Silvia, 2005, 2014).

Against this background of concepts in experimental aesthetics, it is clear the identification of objective image properties in computational aesthetics can provide an important basis for the understanding of aesthetic perception. Indeed, the notion that aesthetic stimuli are endowed by objectively measurable properties that can be universally recognized and are preferred by humans across cultures seems implicit in many studies in computational aesthetics. However, the knowledge about other factors that depend on the cultural context of individual artworks, on the intentions of the artists and on the cognitive and emotional state of the beholder should make us cautious when confronted with claims that particular image properties are universally preferred across individuals, groups of people or cultures.

A major research topic of experimental aesthetics is the investigation of the specific properties of artworks. This research allows us to gain insight into how aesthetic perception is linked to human vision and contributes to our knowledge on how we perceive the world (Graham and Redies, 2010). In the field of experimental aesthetics, researchers have studied a wide variety of aesthetic experiences, ranging from deeply moving emotions elicited when viewing famous artworks in a prestigious museum, to aesthetic ratings of artworks in a laboratory setting, and to visual preferences for simple artificial patterns displayed on a computer screen. This wide range of aesthetic experiences brings up two issues. First, beyond statistical image properties, cultural, social and psychological factors play an important role in aesthetic experience. Undoubtedly, these factors interact with image properties that characterize artworks. Second, the role of specific image properties may depend on the type (or the intensity) of the aesthetic experience studied. For example, if an image property plays a role in aesthetic preference of simple, computer-generated patterns in a laboratory experiment, the same property may not necessarily influence the aesthetic appreciation of high-quality artworks in a museum (or the classification of photographs in a computational study). With these caveats in mind, we will describe several image properties that have been associated with aesthetic experience in the following sections. Again, we do not strive for completeness, but rather review selected examples that seem particularly instructive, with a focus on artworks and photographs.

### 3.2. Luminance and color statistics

The distribution of luminance, color and contrast belong to the low-level image properties that can affect the preference ratings of photographs. For example, Graham and Field (2008) showed that luminance statistics differ between artworks and natural scenes, as do their optical properties. By manipulating luminance statistics in a variety of natural images, including artistic photographs of landscapes, Graham et al. (2016) found that humans prefer images of low skewness (i.e., the third statistical moment) of their luminance distribution, with roughly equal proportions of light and dark in the images. Indeed, artworks tend to have lower-skew luminance histograms than photographs of real scenes across cultures and time periods (Graham and Field, 2007). The authors argue that artists use a non-linear compression to obtain low skewness in their paintings because images with this property can be more efficiently processed by the visual system.

Color is a feature that has been frequently used in classifiers in the field of computational aesthetics (see section 2.1.1). Although it is clear that color contributes much to aesthetics of visual art, there have been relatively few studies on color in experimental aesthetics. For example, by manipulating color statistics of Renaissance paintings, Pinto et al. (2006) studied lighting conditions that viewers consider optimal; they found that human observers generally prefer illumination conditions that yield increased chromatic diversity. Palmer and Schloss (2010) studied human aesthetic preferences for color, using simple visual stimuli. In their ecological valence theory, they suggest that color preferences arise from the affective responses to color-associated objects. In other words, people like colors that are associated with objects they like. In how far these results generalize to artworks remains unclear. Mallon et al. (2014) observed that participants preferred specific combinations of color measures in abstract artworks and that this aesthetic preference is subject to short-term visual adaptation.

In the field of computational aesthetics, Leykin and Cutzu (2003) compared the occurrence of color and luminance intensity edges in paintings and photographs of real scenes. Their results indicated that, in paintings, there are significantly more color-only edges than in photographs of real scenes. Moreover, color edges and intensity edges tend to coincide less frequently in paintings than in photographs of real scenes. Cutzu et al. (2005) build a classifier that combined color, edge and texture properties and distinguished artworks and photographs with 90% accuracy.

Aragón et al. (2008) studied the distribution of luminance in Vincent van Gogh's “Starry Night” and other paintings by the artist. Interestingly, the distribution of luminance fluctuations in some of these images resembled the mathematical distribution of fluid turbulence, as described by the Russian mathematician Andrei Kolmogorov. The authors speculated that the painter might have unwittingly introduced this property in order to produce a special feeling of unease and motion.

### 3.3. Complexity

Complexity relates the subjective impression of how many pictorial elements are contained in a visual stimulus. This property has been studied extensively, both in computational aesthetics and in psychological experiments. Complexity has been captured by a multitude of statistical measures, such as the number of visual elements in an image (Birkhoff, 1933), the fractal dimension (Mureika, 2005; Taylor et al., 2011), GIF compression (Forsythe et al., 2011), overall luminance gradient strength (Braun et al., 2013), or edge density (Redies et al., 2017).

In his seminal work on aesthetics, Berlyne (1974) suggested that images with an intermediate degree of complexity are preferred by humans over images of low or high complexity. His interpretation of the inverted u-shaped relation between beauty and complexity was that preference and interest increase steadily with visual complexity until a maximal level of affective appraisal is reached. With a further increase in complexity, appraisal decreases again because of decreasing preference. Others have argued that humans prefer an intermediate visual complexity because our ancestors lived in a savanna-type landscape of similar complexity (for a review, see Forsythe et al., 2011). The relationship between liking and stimulus complexity is subject to considerable interindividual variability, at least for artificial images (Jacobsen and Höfel, 2002). By automatically clustering the participants, Güçlütürk et al. (2016) described that, for one group of participants, liking decreased as stimuli became more complex, while another group exhibited the opposite pattern of preference (i.e., higher liking for more complex stimuli). Bies et al. (2016a) obtained similar results by investigating preference ratings for exact (mathematical) fractal patterns. They also described that their measure of complexity (fractal dimension) interacted with symmetry and recursion of their stimuli.

Rigau et al. (2008) took Birkhoff's aforementioned idea of aesthetics being a trade off between order and complexity, and proposed different global measures based on principles from information theory and Kolmogorov complexity. The authors applied these measures to nine paintings by van Gogh, Seurat, and Mondrian.

### 3.4. Symmetry, balance and the rule of thirds

Symmetry is a well-established property that plays a prominent role in the perception of many natural and artificial patterns. Symmetry can be perceived at a glance and can affect visual detection, attention, eye movements and physiological arousal (Locher and Nodine, 1989). Not surprisingly, several studies have demonstrated that symmetry is involved also in aesthetic perception. A particularly well-known example is the perception of attractiveness of human faces (Grammer and Thornhill, 1994). In simple geometrical (graphic) and ornamental patterns, symmetry was shown to have a high correlation with aesthetic judgements (Jacobsen and Höfel, 2002; Westphal-Fitch et al., 2013; Rampone et al., 2016; al Rifaie et al., 2017). However, the role of symmetry in photography and artworks seems less clear. The visitor to any art museum will readily realize that simple types of geometrical symmetry (reflectional, translational or rotational) are not general principles of composition in traditional visual art, although symmetry can attract attention if present in a painting (Locher and Nodine, 1989). Accordingly, studies that link symmetry to the aesthetic appreciation of artworks are infrequent (Osborne, 1986). It has therefore been suggested that the link between symmetry and attractiveness/beauty is domain-specific (Little, 2014).

The century-old concept of pictorial balance is related to symmetry, but on a more complex level. Unlike symmetry, it is considered to be an important and universal factor that contributes to the aesthetic appreciation of most types of images, including abstract visual patterns, photographs and artworks (McManus et al., 1985; Gershoni and Hochstein, 2011; Jahanian et al., 2015). According to Arnheim's Gestalt theory of visual balance (Arnheim, 1954), an image is balanced if the center of the displayed attractions is placed on any of the major axes of the image (vertical, horizontal and diagonal). There are different ways to measure balance. For example, in their study on Arnheim's theory, McManus et al. (2011a) used a physicalist approach and measured the center-of-mass of the luminance values in images. They considered an image more balanced if the center-of-mass was closer to the geometrical center of an image. Overall, the authors did not find evidence to support Arnheim's theory when they compared art photographs to photographs that were randomly taken, or when they studied simple geometrical figures. Jahanian et al. (2015) took another approach and modeled pictorial balance in terms of the visual weight of several low-level visual features that are used to calculate visual saliency. In a large set of 120,000 images that were rated highly, the saliency-based image hotspots aligned with Arnheim's axes, thus confirming his theory. A similar difference was obtained in a study on photographic cropping. The details of photographs that were preferred during cropping showed a more balanced saliency distribution than the details that were avoided during cropping (Abeln et al., 2016); no such difference was observed for luminance-based balance McManus et al. (2011b). Some of the computer algorithms that predict ratings of photographs and artworks (see section 2.1.1) incorporate measures of pictorial balance in their calculations (for example, see Ke et al., 2006; Li and Chen, 2009).

The rule of thirds, which is a principle of composition avidly followed in photography, seems to contradict the notion that the major axis of an image play a significant role in balance; it stipulates that salient compositional elements are to be placed close to one of the third lines of the image in order for images to be aesthetically pleasing. The rule of thirds has been used in many computational methods to predict ratings of photographs and artworks (for example, see Datta et al., 2006; Luo and Tang, 2008; Li and Chen, 2009). However, experimental studies did not confirm the significance of this rule in high-quality photographs (Amirshahi et al., 2014a) or “selfie” photographs (Bruno et al., 2014).

### 3.5. Fourier spectral properties

Graham and Field (2007) and Redies et al. (2007b) compared the Fourier spectral properties of natural scenes and images of Western artworks. They found that both types of stimuli share a scale-invariant amplitude (or power) frequency spectrum and both have a similar slope in log-log plots. Similar results were obtained for artworks of East Asian provenance (Graham and Field, 2008) and for other visual stimuli that were created to please the human eye, such as cartoons, comics and mangas (Koch et al., 2010). In contrast, several types of non-art images, such as photographs of simple objects and plants, do not possess this property (Redies et al., 2007b). Notably, photographs of faces portraits have steeper slopes of the log-log plots than human portraits drawn by artists (Redies et al., 2007a). Mather (2014) compared the spectral slopes of 31 artworks with those of closely matching photographs. He found that artists compress the spectral slopes of their works to a relatively narrow range compared to the slopes of the photographs and proposed that the artist's visual system plays a central role in adjusting the spectral slope of artworks. Humans observers tend to prefer artificial, random-phase patterns with Fourier properties similar to natural scenes (Menzel et al., 2015), but exhibit significant interindividual differences in this preference (Spehar et al., 2016). Moreover, the visual preference for these synthetic noise images correlated well with the discrimination sensitivity of the observers for different amplitude spectra of the images (Spehar et al., 2016).

Interestingly, the amplitude spectrum of many uncomfortable visual stimuli contains an excessive energy at medium spatial frequencies and thereby deviates from the linear spectral properties of natural scenes and images of artworks that are perceived as pleasant (Fernandez and Wilkins, 2008; O'Hare and Hibbard, 2011). The Fourier spectral slope of images correlates with measures of image complexity (Table S1 in Redies et al., 2017), in particular with the fractal dimension (Bies et al., 2016b). A shallower slope indicates more power in the high-frequency part of the spectrum; consequently, the images show more fine detail and thus higher complexity.

Schweinhart and Essock (2013) analyzed the Fourier spectral properties in landscape paintings that were produced by a group of local artists, and compared them to photographs of the scenes, which the artists had painted. They asked whether the well-known oblique effect can be observed in paintings. The oblique effect refers to the fact that, in our natural environment, cardinal (horizontal and vertical) edge orientations are more prominent than oblique orientations. In the Fourier domain, this difference translates into stronger amplitudes for cardinal vs. oblique orientations. In the natural environment, this effect is observed only for the lowest spatial frequencies but not for high spatial frequencies. However, the artists implemented the oblique effect also at high spatial frequencies, thus overregulating this image property in their works.

### 3.6. Fractals and self-similarity

The work of the abstract expressionist artist Jackson Pollock (1912–1956) has received particular interest from the scientific community. Taylor performed a fractal analysis of the artist's drip paintings using a box-counting approach and found that Pollock's paintings are not chaotic but possess a fractal structure (Taylor, 2002). This surprising finding prompted a series of investigations of human responses to fractals, which are not only prevalent in nature but can also be found in geometric and mathematical patterns produced by humans. The studies included behavioral investigations, studies of physiological responses, eye tracking and brain imaging studies (Taylor et al., 2011; Taylor and Spehar, 2016). Converging evidence from these studies indicate that both natural and artificial fractals of mid-range complexity (as measured by the fractal dimension) elicit favorable physiological responses and are thus preferred by human observers (see also section 3.3). Fractals have even been shown to reduce stress levels in the observers (Taylor, 2006) and it has been suggested that the beneficial effect of fractal patterns can enhance architecture and our urban environment (Joye, 2007). However, as already observed by Aks and Sprott in their seminal study on chaotic visual patterns (Aks and Sprott, 1996), there are large interindividual differences in human responses to fractals and their complexity (see section 3.3). Interestingly, Pollock created fractal structure in his artworks long before fractal geometry was described and studied in detail in the 1970ies (Mandelbrot and Pignoni, 1983); he must have followed this principle intuitively and without explicit cognitive control. As noted by Alvarez-Ramirez et al. (2008), the finding that Pollock's drip paintings possess fractal structure is closely related to its scale-invariant spectral properties (see section 3.5).

The fractal-like structure of artworks was studied also by Amirshahi et al. (2012) who derived a measure for self-similarity in images, based on a Pyramid Histogram of Oriented Gradients (PHOG) representation of images (Bosch et al., 2007). In this approach, images are self-similar if the Histograms of Oriented Gradients (HOGs) of parts of an image resemble the HOG of the entire image. Redies et al. (2012) applied this measure to different image categories, ranging from natural scenes to man-made stimuli and artworks, including a large and diverse sets of traditional paintings of Western provenance (Amirshahi et al., 2014b). For artworks and most natural patterns, Redies and colleagues reported an intermediate to high self-similarity, whereas other patterns, such as images of simple objects, faces of buildings, were less self-similar.

Both lines of evidence suggest that traditional artworks share specific stimulus properties with our natural environment. Our visual system has adapted to these properties in evolution so that it can process them with a sparse (efficient) code in order to save computational and metabolic resources (Simoncelli and Olshausen, 2001). It has therefore been suggested that artworks are created so that they can be processed efficiently/sparsely by the human visual system (Redies, 2007; Renoult et al., 2016). The concept of sparse coding is familiar also to researchers in computer vision (Mairal et al., 2014). Akin to the efficient coding hypothesis is the idea that artworks can be processed fluently and therefore evoke a pleasant feeling in human observers (Reber et al., 2004). The fluency concept has its origin in the field of psychology; the underlying neuronal mechanism and possible coding strategies in the human brain remain unspecified to date.

### 3.7. Regularities in the orientation of luminance gradients, edges, and lines

In a study on large subsets of traditional Western artworks, histograms of oriented gradients (HOGs; see section 3.6) were found to possess a surprising regularity (Redies et al., 2012; Braun et al., 2013): Artworks possess a relatively uniform spectrum of luminance gradient (edge) orientations. This result implies that all edge orientations in the artworks tend to be similarly prominent. In other words, anisotropy of edge orientations is low in artworks. Other types of images with low anisotropy can be found in nature (for example, large vista scenes and images of plants, lichen growth patterns, branches and clouds; Redies et al., 2012). Anisotropy is larger in images of simple objects, including faces, and other man-made patterns, such as advertisements, building facades and urban scenes, due to the relative prominence of single or a few orientations. For example, horizontal and vertical orientations predominate in images of building facades.

The finding of low anisotropy of edge orientations in artworks was recently confirmed and extended by Redies et al. (2017), who studied edge orientations in different categories of images, including traditional artworks of different cultural provenance (Western, Islamic and East Asian). They showed that the art images possess a more uniform histogram of edge orientations across cultures than many non-art types of images, in particular, photographs of man-made objects and scenes. This result mirrors the low anisotropy found in artworks (see above). In addition, by pairwise comparison of edge orientations across each image, Redies and colleagues found that edge orientations are independent of each other across art images, except for edge pairs at short distances, which tend to be collinear. In other words, the edge orientation at one position of an image does not allow predicting the orientations of distant edges at other positions in the same image. Similar statistical regularities of edge orientations are observed in some natural images, such as lichen growth patterns. This property is independent of cultural provenance, artistic genre or technique, or image content of the artworks studied. The authors speculated that this regularity might relate to the notion of “good composition” (Arnheim, 1954) or “visual rightness” (Locher et al., 1999), which has been advanced for traditional artworks.

Another regularity with respect to the perception of contours is that smoothly curved lines and objects are generally preferred over sharply angular ones (Gómez-Puerto et al., 2015). Interestingly, humans share this preferences not only across cultures but also with great apes (Munar et al., 2015). As a possible explanation, Bar and Neta (2006) proposed that sharp transitions in contour convey a sense of threat in the observer and are therefore disliked. However, Bertamini et al. (2016) questioned this notion and provided experimental evidence that humans prefer curvature due to its intrinsic characteristics and not because they reject the threat potential of angular contours.

## 4. Conclusion and outlook

In recent years, computer vision has successfully contributed computational methods to the evaluation of photographs and digitally reproduced artworks. In the present work, we discussed recent progress in this field, which has become known as computational aesthetics. Specifically, we reviewed methods that were developed to predict the aesthetic rating of photographs and artworks by computational approaches. For artworks, we provided an overview on applications of computational algorithms to artist identification, style prediction, art historical questions, and forgery detection.

In general, researchers in the computer vision community tend to measure success by comparing different methods regarding their accuracy of classification or prediction. When using the same database, systems can easily be compared and finding the best working approach is straightforward. However, with recent advances in technology, algorithmic and larger datasets, the best-performing classifiers have become black boxes and their discrimination boundaries are no longer obvious. From an application standpoint of view, this is not necessarily a limitation. For example, such systems can be readily deployed in image processing pipelines to identify images of high vs. low aesthetic value. While early methods where restricted to the formal aspects of a scene, more advanced methods, like Deep Neural Networks, can take into account the content of images as well. It was shown that the inclusion of content results in major improvements, because different stylistic elements come along with different content matter. For example, bright colors are usually more pronounced in pleasant images that depict fresh fruits than in gloomy images of street scenes at night. Such combinatorial information can improve classification results.

Lately, computational methods have gained increasing popularity also in the field of experimental aesthetics, an area of research that has a long tradition as a branch of psychology and, more recently, of neuroscience. In experimental aesthetics, the focus is not on improving algorithms for rating prediction systems or identifying artists or artistic styles, but rather on gaining a better understanding of what specific stimulus properties induce human observers to reach judgements on beauty and to have an aesthetic experience. For example, as discussed in section 3, converging evidence suggests that some global image properties that also characterize natural scenes can be found in large subsets of traditional artworks.

With recent developments in Deep Learning, it has become harder to share knowledge between computational aesthetics and experimental aesthetics. In the early days, insights from the active field of experimental aesthetics provided a wealth of knowledge, also for computational aesthetics. This knowledge resulted in the development of computational algorithms based on handcrafted features, which were known (or suspected) to contribute to the aesthetic appeal of an image. During this time, empirical aesthetics also profited greatly from the computational methods because, for the first time, very large datasets of images could be analyzed, rather than the small number of images that are usually tested in psychological experiments with human observers. However, with Deep Learning, it has became harder for empirical aesthetics to catch up with the computational approaches. Deep Learning models basically represent black boxes, which prevent insight into what features they learn and how they use them to evaluate the aesthetic quality of images, which is the main motivation for empirical aesthetics. In future work, it will therefore be essential to gain a better understanding and interpretability of the decision boundaries that the computational models draw, in order to identify concrete properties of human aesthetic preference. Moreover, recent generative models from computer vision (Gatys et al., 2016) are capable of producing synthetic images that match the style of famous painters, and are no longer discriminative only. This generative approach may provide researchers with well-controlled stimuli for testing human observers in experimental aesthetics.

In conclusion, much can be learned if the two areas of aesthetic research can be recombined, taking advantage of the methodological advances in computational aesthetics and the identification of perceptual mechanisms in experimental aesthetics. As an example, we recently investigated the variability of CNN feature responses to traditional artworks and non-art images and found that the two categories of images can be separated by a classifier that is based on only two variance values (Brachmann et al., 2017). However, results for some styles of (post-)modern and contemporary art clearly deviated from traditional art. The investigation of differences between art styles may therefore be of particular interest in the future, not only in computational aesthetics but also in experimental aesthetics. Moreover, in view of the interindividual differences in aesthetic preferences (see section 3.1), cultural diversity will be an important issue in future research.

## Author contributions

AB and CR conceived this review, carried out the literature search and wrote the manuscript.

### Conflict of interest statement

The authors declare that the research was conducted in the absence of any commercial or financial relationships that could be construed as a potential conflict of interest.
